# Tobacco-associated carcinogenesis with nicotine-mediated tumor promotion across the gastrointestinal tract: A narrative review

**DOI:** 10.18332/tpc/225663

**Published:** 2026-09-28

**Authors:** Sanyukta Padmanabhan, Mariyah Khan-Garrard, Bhavya Gupta, Kevin A. Carnevale

**Affiliations:** 1 Des Moines University West Des Moines United States

**Keywords:** nicotine, gastrointestinal neoplasms, tobacco exposure, nicotinic acetylcholine receptors, nitrosamines

## Abstract

Nicotine exposure remains prevalent through conventional tobacco products and emerging nicotine delivery systems. Although nicotine is not classified as a classical direct genotoxic carcinogen, accumulating evidence suggests that it functions as a tumor-promoting modulator within tobacco-associated carcinogenesis. While individual studies have described organ-specific effects of nicotine within the gastrointestinal (GI) tract, a consolidated synthesis of nicotine-associated mechanisms across GI organ systems has been limited. The aim of this narrative review is to summarize the relevant nicotine-driven pathways and risk factors involved in GI neoplasms. A structured literature search was conducted using PubMed and Google Scholar to identify studies evaluating nicotine-associated carcinogenic mechanisms within the gastrointestinal tract. Four reviewers screened articles after 1993 for relevance to nicotine-mediated gastrointestinal tumorigenesis. Human epidemiological studies, meta-analyses, animal models, and original research were included to capture both clinical associations and molecular signaling pathways. The final search was performed in December 2025. Articles within the literature search describe how nicotine and nicotine-associated signaling influence tumorigenesis throughout the gastrointestinal tract. Across tissues, nicotine activates nicotinic acetylcholine receptors (nAChRs) and downstream signaling cascades that regulate cellular proliferation, apoptosis resistance, angiogenesis, and inflammatory responses. Common pathways include MAPK/ERK, PI3K/AKT, and JAK/STAT signaling, although organ-specific vulnerabilities modify these effects. Tobacco-specific nitrosamines contribute to mutagenesis in oral and pancreatic tissues; YAP/Hippo signaling is implicated in esophageal carcinogenesis; KRAS-associated adenoma–carcinoma progression influences colorectal and pancreatic malignancies; and pro-angiogenic COX-2–mediated pathways support gastric and hepatobiliary tumor growth. Nicotine-associated immune modulation may further facilitate virus-related malignancies in select contexts. Collectively, available data suggest that nicotine acts as a context-dependent tumor promoter across gastrointestinal tissues by amplifying proliferative and inflammatory signaling within established carcinogenic frameworks. By synthesizing molecular and epidemiological findings across GI sites, this review provides a unified framework for understanding nicotine-associated contributions to gastrointestinal tumorigenesis and highlights areas requiring further investigation.

## Introduction

While cigarette smoking in the United States has declined steadily since the mid-1960s, dropping to 11.6% by 2022 and continuing to fall through 2025, overall nicotine consumption has shifted rather than disappeared^1^. The explosive popularity of electronic nicotine delivery systems (vapes) and oral nicotine pouches, whose sales nearly tripled between 2023 and 2024, has caused nicotine prevalence in the United States to remain largely unchanged since 2017^1^. This shift has resulted in sustained systemic exposure, particularly among young adults, through ‘dual-use’ patterns that bypass traditional combustible tobacco-control metrics^1^.

Nicotine promotes gastrointestinal (GI) carcinogenesis through several conserved mechanisms across multiple organ sites. Key proliferative pathways include MAPK/ERK, which regulates cell growth and differentiation; PI3K/AKT, which promotes cell survival and inhibits apoptosis; and JAK/STAT, which transduces cytokine signals to drive proliferation, survival, and immune evasion^2^. In addition, specific oncogenic drivers are implicated in certain cancers, such as K-RAS mutations in pancreatic cancer, APC hypermethylation in colorectal cancer that accelerates the adenoma–carcinoma sequence, and YAP/Hippo pathway activation in esophageal and small intestinal cancers that promotes uncontrolled cell proliferation^2,3^. While these signaling pathways are commonly activated by nicotine across the gastrointestinal tract, each organ exhibits unique vulnerabilities that influence tumor initiation and progression^3^.

Despite the well-documented mutagenic risks of tobacco smoke, a consolidated synthesis of nicotine’s independent role as a systemic tumor promoter within the gastrointestinal tract remains limited. This review links nicotine-associated signaling to gastrointestinal cancer by integrating shared molecular mechanisms with organ-specific features. The aim is to highlight nicotine as a critical, context-dependent driver of cancer progression in the modern era of diverse nicotine delivery.

This is a structured narrative review examining molecular mechanisms and organ-specific pathways through which nicotine contributes to gastrointestinal tumorigenesis. A structured literature search was conducted using PubMed and Google Scholar to identify studies evaluating nicotine-associated carcinogenic mechanisms within the gastrointestinal tract. The final search was performed in December 2025. Search terms included combinations of keywords such as ‘nicotine’, ‘gastrointestinal’, ‘tumorigenesis’, ‘nAChR’, ‘Barrett’s esophagus’, ‘hepatocellular carcinoma’, and ‘adenoma–carcinoma sequence’. Boolean operators (AND, OR) were used to refine search combinations. Reference lists of relevant articles were manually screened to identify additional primary studies. All retrieved citations were imported into Zotero reference management software, and duplicates were removed prior to screening.

Studies were eligible if they examined the role of nicotine or nicotine-associated metabolites in gastrointestinal carcinogenesis. Human epidemiological studies, animal models, and *in vitro* mechanistic investigations were included to capture both clinical associations and molecular signaling pathways. Original research articles were eligible. Published meta-analyses were included when relevant to epidemiological associations between nicotine exposure and gastrointestinal malignancies. When studies cited within meta-analyses were deemed mechanistically relevant, the original primary sources were reviewed and incorporated as appropriate. Editorials, commentaries, and non-research articles were excluded. Only studies published in English were included. No explicit lower date restriction was applied; the earliest included study was published in 1993. Unpublished studies and conference abstracts were not included. Four reviewers screened titles and abstracts for relevance to predefined thematic domains related to nicotine-mediated gastrointestinal tumorigenesis. Full-text articles were independently assessed by three reviewers. Inclusion decisions were based on relevance to mechanistic pathways, organ-specific carcinogenic processes, or epidemiological associations. Disagreements were resolved through discussion and unanimous consensus. Three reviewers independently extracted key study characteristics using a standardized framework. Extracted domains included study design, exposure characteristics, molecular signaling pathways, enzymatic mechanisms, and organ-specific oncogenic outcomes. When necessary, reviewers collaborated to determine appropriate integration of findings into the narrative structure. Given the heterogeneity of study designs, populations, and mechanistic endpoints, quantitative meta-analysis was not performed. Findings were synthesized narratively and organized by gastrointestinal organ system and shared oncogenic signaling pathways. Formal risk-of-bias assessment and certainty-of-evidence frameworks were not applied due to the narrative and mechanistic scope of this review and the inclusion of heterogeneous human, animal, and *in vitro* studies.

## Commentary

Formation of GI neoplasms has different risks, susceptibility, and molecular pathways that are stimulated by nicotine, tobacco, and/or its metabolites, related to these substances.

Table 1 summarizes the mechanisms of different neoplasms according to location within the GI tract.

**Table 1:** Unique mechanisms of nicotine-driven tumorigenesis across gastrointestinal cancers

Cancer type	Unique mechanisms	References
Oral squamous cell carcinoma (OSCC)	Direct mucosal exposure to tobacco-specific nitrosamines (NNK, NNN); DNA adduct formation via CYP450 metabolism; leukoplakia as a premalignant lesion; progression modulated by smoking cessation	4,5
Esophageal adenocarcinoma	Chronic GERD → intestinal metaplasia; acid-induced dysplasia; specific to the distal esophagus	7-9
Esophageal squamous cell carcinoma	YAP-1 / Hippo pathway activation; proto-oncogene dysregulation; tobacco/alcohol synergism	10,11
Gastric cancer	β-adrenergic receptor involvement; COX-2 / PGE2 upregulation; VEGF-mediated angiogenesis; sex-specific susceptibility	13-15
Pancreatic cancer	Tobacco-specific nitrosamines reach pancreas → DNA adducts → early K-RAS mutations; effect depends on tobacco type	16,18
Biliary tract cancer	Fibrosis and cholangiocyte proliferation; nitrosamine exposure via bile; ERK1/2-mediated profibrotic gene expression; subsite-specific susceptibility	19-24
Hepatocellular carcinoma	Enhanced stem-like properties; EMT induction; synergy with viral hepatitis, alcohol, or metabolic dysfunction	26-29
Small-intestinal cancer	Altered intestinal stem cell proliferation; microbiome modulation; rare tumor types (carcinoids, neuroendocrine)	30-33
Colorectal cancer	APC hypermethylation → adenoma–carcinoma sequence; inflammation–dysplasia–carcinoma axis modulation	34,36-39
Anal cancer	HPV-mediated oncogenesis; nicotine-induced immune suppression → persistent HPV infection; oxidative stress enhancing viral transformation	40-43

Search of the literature in 2025 dating back to 1993 of nicotine-associated carcinogenic mechanisms within the gastrointestinal tract. Human epidemiological studies, animal models, and *in vitro* mechanistic investigations were included to capture both clinical associations and molecular signaling pathways. Original research articles were reviewed. Also, published meta-analyses were included when relevant to epidemiological associations between nicotine exposure and gastrointestinal malignancies.

### Oral squamous cell carcinoma and its precancerous lesion, oral leukoplakia

Tobacco exposure is strongly linked with oral squamous cell carcinoma (OSCC) across all regions of the oral cavity, including the tongue, floor of the mouth, and gingiva. A common precursor is oral leukoplakia, a white mucosal plaque observed in up to 30% of chronic smokers and considered a premalignant lesion arising from sustained epithelial irritation and hyperproliferation. Longitudinal studies indicate that approximately 30% of leukoplakic lesions in smokers progress to oral cancer over time^4,5^. Importantly, leukoplakia may partially or completely regress with smoking cessation, whereas continued or chronic tobacco use increases the likelihood of irreversible dysplastic changes and progression to OSCC. Oral carcinogenesis is unique in that it is strongly influenced by direct mucosal exposure to tobacco-specific nitrosamines, particularly 4-(methylnitrosamino)−1-(3-pyridyl)−1-butanone (NNK) and N'-nitrosonornicotine (NNN), which accumulate at high concentrations within the oral mucosa^6^. These compounds undergo CYP450-mediated metabolic activation, forming DNA-reactive intermediates that generate DNA adducts, covalent chemical modifications of DNA that disrupt normal base pairing and replication fidelity, thereby promoting mutagenesis, genomic instability, and malignant transformation^6^.

While nicotine itself is not classified as directly carcinogenic, it may function as a tumor promoter by enhancing proliferative signaling and sustaining chronic inflammation, thereby facilitating the clonal expansion of genetically altered epithelial cells and progression to OSCC^6^.

### Esophageal cancer: nicotine-driven pathways

In the esophagus, current and prior history of smoking is associated with a higher rate of esophageal squamous cell carcinoma (ESCC)^7^. Additionally, smoking also correlates with esophageal adenocarcinoma, with a large meta-analysis with over 12000 participants showing an increased rate of adenocarcinoma of the esophagus with a strong dose-dependent association^8^. Worldwide tobacco-related esophageal cancer is highest in India and China^7^.

Esophageal adenocarcinomas are commonly linked with chronic gastroesophageal reflux disease (GERD). A well-known pathway identifies nicotine as impacting the lower esophageal sphincter pressure, increasing stomach acid backflow into the distal one-third of the esophagus, causing GERD symptoms^8,9^. Persistent acid exposure to this area induces gastric mucosa metaplasia. Over time, chronic metaplasia can progress to dysplasia, better known as Barrett’s esophagus, and ultimately raises the risk for the development of adenocarcinoma^9^.

In contrast, ESCC demonstrates a strong association with tobacco exposure, with a 7-fold increased risk among smokers. A cell line experiment performed by Zhao et al.^10^ suggests that the mechanism of squamous cell carcinoma involves the activation of Yes-associated protein 1 (YAP-1), a key transcription factor in the ‘mammalian Hippo’ pathway, functioning as an oncogenic factor for many cancers, including esophageal cancer. Nicotine administration has been shown to induce the YAP-1 pathway, suggesting a link to the development of squamous cell carcinoma^11^. Additionally, tobacco smoke contains at least 70 recognized carcinogens, including tobacco-specific nitrosamines, polycyclic aromatic hydrocarbons like benzopyrene, benzene, formaldehyde, and arsenic that can cause cancer by damaging DNA, inducing mutations, and promoting tumor growth^11^.

### Gastric cancer: COX-2 and receptor-mediated effects

Tobacco smoking is a well-established risk factor for both the development and progression of gastric cancer^12^. Gastric cancer rates worldwide related to smoking are decreasing overall; however, there is a higher incidence in regions such as northern Africa, western Sub-Saharan Africa, the Middle East, eastern Europe, and the Caribbean^12^. Large epidemiological studies demonstrate a 1–2.5-fold increased risk of gastric carcinoma among smokers compared with non-smokers, with a consistently stronger association observed in males^12^. Nicotine has been shown to amplify gastric carcinogenesis through activation of nAChRs and β-adrenergic receptors (β-ARs) expressed on gastric epithelial and tumor cells. Engagement of these receptors stimulates multiple oncogenic signaling cascades, including the MAPK/ERK, PI3K/AKT, and JAK/STAT pathways, which collectively enhance cellular proliferation, migration, invasion, and resistance to apoptosis^13^.

A central downstream effector of nicotine signaling involves cyclooxygenase-2 (COX-2), an inducible enzyme frequently overexpressed in gastric malignancies. Nicotine exposure has been shown to upregulate COX-2, resulting in increased prostaglandin E2 (PGE2) production and subsequent enhanced tumor cell survival and stimulation of angiogenesis through vascular endothelial growth factor (VEGF)^14^. Experimental models of chronic nicotine exposure demonstrate this pathway of accelerated tumor growth and increased microvascular density, highlighting the importance of the ERK/COX-2/VEGF axis in nicotine-driven gastric tumorigenesis^15^.

### Pancreatic cancer: α7-nAChR signaling and DNA adduct formation

The association between nicotine exposure and pancreatic cancer appears to be dependent on the route of exposure. Combustible cigarette smoking is associated with a significantly increased risk of pancreatic cancer, with current smokers demonstrating approximately 70% higher odds of pancreatic cancer compared with never smokers in an observational cohort^16^. In contrast, large epidemiological studies consistently demonstrate no significant association between smokeless tobacco, including snus, and pancreatic cancer risk, with multiple nationally representative and pooled cohort analyses reporting no excess pancreatic cancer mortality or incidence among smokeless tobacco users^17^.

Mechanistically, tobacco carcinogens reach pancreatic tissue and undergo metabolic activation, forming DNA adducts such as O6-methylguanine. These adducts induce oncogenic mutations, particularly mutations in K-RAS, which is one of the earlier events in pancreatic carcinogenesis^18^. These carcinogenic effects may be further amplified by interactions with chronic inflammation, fibrosis, and other environmental or metabolic stressors within the pancreatic microenvironment^18^.

### Biliary tract cancer: fibrosis, nitrosamines, and α7-nAChR signaling

Biliary tract cancers represent a heterogeneous group of malignancies with distinct anatomic and etiologic profiles. Biliary tract cancers account for approximately 3% of all gastrointestinal malignancies and are generally rare but clinically aggressive neoplasms worldwide^19^. Large meta-analyses and pooled cohort studies have demonstrated an association of tobacco use with extrahepatic bile duct and ampulla of Vater cancers, but no causal link with gallbladder cancers themselves^20^. Notably, some case–control and population-based studies have reported an increased risk of gallbladder cancer among chronic smokers, although these associations appear variable across populations and may reflect broader tobacco exposure rather than nicotine alone^21^. These contradicting studies highlight the inconsistent evidence regarding tobacco’s role in gallbladder carcinogenesis. Tobacco-mediated carcinogenic pathways may differentially affect biliary epithelial cells, supporting the concept of distinct molecular vulnerabilities across biliary tract subsites^21^. Beyond epidemiological associations, cellular models indicate that nicotine itself may actively promote biliary epithelial proliferation and tumor progression. Chronic nicotine exposure stimulates cholangiocyte proliferation and fibrotic remodeling through activation of α7-nAChRs expressed on biliary epithelial cells^22^. Human cholangiocarcinoma cell lines exhibit elevated α7-nAChR expression, and nicotine exposure enhances ERK1/2 phosphorylation, cell proliferation, and survival^23^. Consistent with this, in rat models receiving continuous nicotine administration via osmotic minipumps, α7-nAChR expression is upregulated on cholangiocyte membranes, leading to Ca2-dependent ERK1/2 activation and increased expression of profibrotic genes such as α-smooth muscle actin and fibronectin, markers associated with biliary fibrosis and premalignant architectural distortion^24^. *In vivo* xenograft models demonstrate that chronic nicotine treatment accelerates cholangiocarcinoma growth and intra-tumoral fibrosis, suggesting that nicotine can contribute to biliary tract carcinogenesis through conserved α7-nAChR–ERK signaling pathways^23^. This sustained proliferative and fibrotic response suggests that chronic nicotine exposure creates a microenvironment characterized by heightened cellular turnover and ductal remodeling that may predispose to malignant transformation^23,24^.

Similar to pancreatic cancer, nitrosamines cause mutations and enhance the initiation of carcinogenesis. In the hepatobiliary context, such metabolites and other reactive tobacco byproducts are excreted into bile, prolonging exposure of biliary epithelial cells to genotoxic compounds and increasing oxidative stress and DNA damage, which creates a mutagenic environment in the bile ducts. Persistent oxidative injury also activates pro-inflammatory signaling pathways (e.g. NF-κB) and upregulates enzymes like COX-2, which are commonly overexpressed in biliary tract cancers and support proliferative and survival signaling^25^. These processes provide a framework through which tobacco exposure contributes to biliary tract malignancy.

### Hepatocellular carcinoma: nicotine-induced oxidative stress and proliferation

Hepatocellular carcinoma (HCC) demonstrates an increased incidence and mortality among smokers, with synergistic risk amplification observed in individuals with chronic hepatitis B or C infection, excessive alcohol consumption, or metabolic dysfunction-associated steatohepatitis^26^. A large case-control study indicates a 2.46 times higher likelihood of developing HCC than people who never smoked.

Mechanistic studies suggest nicotine influences hepatic tumor biology through oxidative stress and receptor-mediated signaling. In hepatocellular cancer cells, nicotine induces CYP1A1 expression via transcription factors including NF-κB, AP-1, and AhR, leading to increased reactive oxygen species (ROS) generation and Akt pathway activation, which promotes cell proliferation and survival^27^. Additionally, nicotine exposure enhances epithelial–mesenchymal transition and stem-like properties, increasing expression of proliferative markers such as Ki-67 and cyclin D1, and promoting invasive behavior in HCC models^28^.

Similar to GI malignancies, nicotine activates α7-nAChRs, which are expressed in normal hepatocytes and HCC cells, promoting proliferation, migration, invasion, and maintenance of tumor-initiating cells phenotypes specifically through the JAK2/STAT3 signaling axis^29^. Additionally, inhibition of α7-nAChR reduces cell viability and invasiveness and downregulates proteins involved in cytoskeletal regulation and extracellular matrix remodeling supporting a role for α7-nAChR contributing to HCC progression^29^.

### Small-intestinal cancer

In contrast to other gastrointestinal malignancies, small intestinal cancers, including adenocarcinoma, neuroendocrine tumors, and carcinoids, are relatively rare, and tobacco-related associations are less well characterized. Epidemiological evidence suggests that smoking is associated with certain small intestinal tumors. A large European population-based study found that ever having smoked was correlated with an elevated risk of small bowel carcinoid tumors, with a 1.9-fold increased odds compared with non-smokers^30^. Similarly, a Utah statewide cohort analysis reported that tobacco exposure was observed with an increased risk of small intestine neuroendocrine tumors independent of family history^31^.

Additionally, registry-based analyses also describe smoking as a modest risk factor for small intestinal adenocarcinomas and neuroendocrine tumors, although results are inconsistent and may be limited by small sample sizes^32^.

Mechanistic studies specific to the small intestine are limited; however, several lines of evidence support a potential link between tobacco exposure and small intestinal tumorigenesis^11,33^. Cigarette smoke has been shown to alter the gut mucosal microbiota, including in the duodenum, reducing bacterial diversity and shifting microbial populations, which may affect local inflammation and epithelial homeostasis in the small intestine, a microenvironmental change that could influence carcinogenic processes^33^.

Separately, translational research in murine models demonstrates that nicotine can enhance the proliferative activity of intestinal stem cells via α7-nAChR–dependent activation of YAP/TAZ and Notch signaling pathways, increasing their tumorigenic potential in the context of tumor suppressor loss (e.g. Apc deficiency)^11^. Together, alterations in mucosal homeostasis and nicotine-driven stem cell signaling provide a potential mechanism through which tobacco exposure may contribute to small intestinal tumorigenesis, although further targeted studies are needed^11^.

### Colorectal cancer: APC hypermethylation and nAChR activation

Colorectal cancer (CRC) has a moderately increased incidence in smokers. A large meta-analysis of smokers showed a 14% increase in the likelihood of developing colorectal cancer in smokers and former smokers compared with never smokers^34^. Observational studies further demonstrate sex- and subsite-specific patterns^35^. Similar to gastric cancer, a large observational study revealed that men have a stronger association with left-sided colon tumors, whereas women show a greater risk for right-sided lesions, suggesting differential susceptibility related to hormonal modulation, microbiome composition, or regional differences in carcinogen exposure^35,36^.

Mechanistic studies indicate that nicotine can influence colonic epithelial biology through receptor-mediated signaling. Nicotine promotes proliferation, migration, and epithelial–mesenchymal transition in colonic epithelial cells via nAChRs and downstream MAPK/ERK and PI3K/AKT signaling pathways^37^. Similar to this, murine models demonstrate that nicotine directly modulates the inflammation–dysplasia–carcinoma axis, reducing cytokine-driven colitis while paradoxically promoting colitis-associated tumorigenesis, indicating a direct effect on inflammatory signaling pathways involved in malignant progression^38^.

The carcinogenic impact of nitrosamines in CRC is largely attributed to early mutations in APC, particularly through hypermethylation of CpG islands, facilitating initiation of the adenoma–carcinoma sequence, and high microsatellite instability^34,39^. While nitrosamines contribute to mutational initiation, these data suggest that nicotine contributes to enhancing the survival and proliferation of premalignant adenomatous cells, contributing to disease progression^37^.

### Anal cancer: nicotine, HPV, and immunomodulation

Epidemiological studies report that tobacco smoking substantially increases anal cancer risk, particularly among premenopausal women, with current smokers exhibiting a more than fivefold higher risk compared with lifelong non-smokers^40,41^. Notably, current smokers with persistent human papillomavirus (HPV) infection have a 74% increased likelihood of developing anal cancer^42^.

Mechanistically, smoking appears to potentiate HPV-mediated oncogenesis rather than acting as a primary mutagenic initiator. Tobacco constituents, including nicotine, have been shown to impair local immune surveillance by reducing antigen presentation, altering cytokine profiles, and suppressing cell-mediated immunity^43^. This immunosuppressive effect facilitates persistent HPV infection and increases the likelihood of viral oncogene (E6 and E7) expression, which inactivates tumor suppressors p53 and Rb^43^.

Tobacco-related oxidative stress may further contribute to genomic instability in HPV-infected epithelial cells, promoting malignant transformation^43^. Although research is limited in nicotine receptor signaling in anal carcinoma, the convergence of immune suppression, chronic inflammation, and viral oncogene activation provides a link with smoking to anal cancer development.

### Interpretation of findings

Nicotine appears to contribute to gastrointestinal carcinogenesis primarily through tumor-promoting mechanisms that enhance proliferative signaling, inflammatory responses, and resistance to cell death. Within this carcinogenic context, nicotine emerges as a biologically active tumor-promoting agent. Although not classified as a direct genotoxic carcinogen, nicotine activates nicotinic acetylcholine receptors (nAChRs) and downstream signaling pathways, including MAPK/ERK, PI3K/AKT, JAK/STAT, and inflammatory mediators such as COX-2^14,29,37,44-46^. Activation of these pathways enhances cellular proliferation, resistance to apoptosis, angiogenesis, epithelial–mesenchymal transition, and stem-like phenotypes^44-47^. Collectively, these signaling effects may amplify tumor growth and progression once genetic alterations have been established.

While shared signaling mechanisms are observed across gastrointestinal tissues, tumor development is shaped by organ-specific biology. In the oral cavity, direct mucosal exposure to nitrosamines promotes DNA damage, with nicotine potentially facilitating proliferative expansion of transformed cells^4,5^. Esophageal malignancies demonstrate interactions between tobacco exposure, reflux-mediated epithelial injury, and YAP/Hippo pathway activation^10,11^. Gastric tumorigenesis appears closely linked to inflammatory and COX-2–mediated signaling, whereas pancreatic and biliary cancers reflect early mutagenic events driven by tobacco carcinogens alongside α7-nAChR proliferative signaling^14,18^. In hepatic and colorectal tissues, nicotine-related oxidative stress and receptor-mediated signaling intersect with established inflammatory and genetic pathways of carcinogenesis^29,34,38,39^.

Tobacco exposure contributes to gastrointestinal carcinogenesis through both mutagenic and tumor-promoting mechanisms. Epidemiological evidence consistently demonstrates increased risk of multiple gastrointestinal malignancies among smokers, with organ- and subsite-specific variability^7,12,18^. The sex disparity with higher rates of esophageal and gastric carcinoma in males is thought to reflect differences in smoking intensity, duration, and hormonal modulation of carcinogenic pathways^7,12^. Across cancer types, tobacco-specific carcinogens, particularly nitrosamines, are strongly implicated in mutational initiation through DNA adduct formation, genomic instability, and early oncogenic alterations such as KRAS mutation, APC dysregulation, and microsatellite instability^18,24,39,44^. These initiating events establish the molecular foundation upon which subsequent tumor progression may occur.

These findings support a model in which tobacco-derived carcinogens initiate gastrointestinal malignancy, while nicotine-associated signaling may function as a context-dependent amplifier of tumor progression^45-47^. This dual framework underscores the importance of distinguishing between mutational initiation and receptor-mediated tumor promotion when evaluating the biological effects of tobacco exposure across gastrointestinal cancers.

### Limitations

Several limitations of the available literature should be considered. Much of the mechanistic evidence derives from experimental models, which may not fully reflect human disease. Epidemiological studies frequently use tobacco exposure as a surrogate for nicotine exposure, making it difficult to isolate nicotine’s independent effects from other carcinogens in tobacco smoke. In addition, certain malignancies, such as small intestinal and biliary tract cancers, remain relatively rare, limiting the availability of large epidemiological studies.

This review also has methodological limitations. This was a narrative review and not systematic. The literature search was limited to two databases and English-language publications, which may have excluded relevant studies leading to publication bias. Additionally, restricting inclusion to studies published after 1993 allowed emphasis on contemporary mechanistic research but may have excluded earlier foundational work. Finally, heterogeneity in study design, exposure models, and outcome measures precluded quantitative meta-analysis, and findings were therefore synthesized narratively. It did not have a systematic framework, such as PICOS (population, intervention, comparison, outcome, and study design), to build a particular literature search strategy.

### Implications

Despite these limitations, several important implications arise. As nicotine delivery methods continue to evolve, particularly with the growing use of electronic cigarettes and other non-combustible products, understanding the biological consequences of nicotine exposure independent of traditional tobacco smoke becomes increasingly important. Even in the absence of many combustion-related carcinogens, nicotine signaling itself may still influence tumor biology. This possibility is particularly relevant for individuals with premalignant gastrointestinal conditions, where pro-proliferative and anti-apoptotic signaling could contribute to disease progression.

Future research should focus on clarifying the long-term cancer risk associated with newer nicotine delivery systems and further defining the roles of specific nAChR subtypes in gastrointestinal tumorigenesis. Additional work examining nicotine interactions with the gut microbiome, immune signaling, and intestinal stem cell regulation may also provide new insights into how nicotine influences cancer development across the gastrointestinal tract.

## Conclusion

Nicotine appears to contribute to gastrointestinal carcinogenesis primarily through tumor-promoting mechanisms that enhance proliferative signaling, inflammatory responses, and resistance to cell death. While the specific pathways involved vary among gastrointestinal organs, some commonality with nicotine acetylcholine receptor-mediated activation represents commonality in tumorigenesis pathways. As patterns of nicotine exposure continue to change worldwide, further research will be essential to better understand its long-term impact on gastrointestinal cancer risk and progression.
